# Integrated omics and machine learning-assisted profiling of cysteine-rich-receptor-like kinases from three peanut spp*.* revealed their role in multiple stresses

**DOI:** 10.3389/fgene.2023.1252020

**Published:** 2023-09-20

**Authors:** Kinza Fatima, Muhammad Sadaqat, Farrukh Azeem, Muhammad Junaid Rao, Norah A. Albekairi, Abdulrahman Alshammari, Muhammad Tahir ul Qamar

**Affiliations:** ^1^ Integrative Omics and Molecular Modeling Laboratory, Department of Bioinformatics and Biotechnology, Government College University Faisalabad (GCUF), Faisalabad, Pakistan; ^2^ State Key Laboratory for Conservation and Utilization of Subtropical Agro-Bioresources, Guangxi Key Laboratory of Sugarcane Biology, College of Agriculture, Guangxi University, Nanning, Guangxi, China; ^3^ Department of Pharmacology and Toxicology, College of Pharmacy, King Saud University, Riyadh, Saudi Arabia

**Keywords:** peanut, pangenome-wide, receptor-like kinases, gene ontology enrichment, abiotic stress, multi-stress-related genes, random forest

## Abstract

*Arachis hypogaea* (peanut) is a leading oil and protein-providing crop with a major food source in many countries. It is mostly grown in tropical regions and is largely affected by abiotic and biotic stresses. Cysteine-rich receptor-like kinases (CRKs) is a family of transmembrane proteins that play important roles in regulating stress-signaling and defense mechanisms, enabling plants to tolerate stress conditions. However, almost no information is available regarding this gene family in *Arachis hypogaea* and its progenitors. This study conducts a pangenome-wide investigation of *A. hypogaea* and its two progenitors, *A. duranensis* and *A. ipaensis CRK* genes (*AhCRKs*, *AdCRKs*, and *AiCRKs*). The gene structure, conserved motif patterns, phylogenetic history, chromosomal distribution, and duplication were studied in detail, showing the intraspecies structural conservation and evolutionary patterns. Promoter *cis*-elements, protein–protein interactions, GO enrichment, and miRNA targets were also predicted, showing their potential functional conservation. Their expression in salt and drought stresses was also comprehensively studied. The CRKs identified were divided into three groups, phylogenetically. The expansion of this gene family in peanuts was caused by both types of duplication: tandem and segmental. Furthermore, positive as well as negative selection pressure directed the duplication process. The peanut *CRK* genes were also enriched in hormones, light, development, and stress-related elements. MicroRNA (miRNA) also targeted the *AhCRK* genes, which suggests the regulatory association of miRNAs in the expression of these genes. Transcriptome datasets showed that *AhCRKs* have varying expression levels under different abiotic stress conditions. Furthermore, the multi-stress responsiveness of the *AhCRK* genes was evaluated using a machine learning-based method, Random Forest (RF) classifier. The 3D structures of *AhCRKs* were also predicted. Our study can be utilized in developing a detailed understanding of the stress regulatory mechanisms of the *CRK* gene family in peanuts and its further studies to improve the genetic makeup of peanuts to thrive better under stress conditions.

## 1 Introduction

Plants are exposed to a great number of biotic as well as abiotic stresses throughout their life. These include salinity, drought, cold, and pathogens. This has led to the evolution of several mechanisms in the immune system that helps them survive during these stresses. One of these mechanisms is receptor-like kinases (RLKs), which belong to transmembrane proteins, which sense external signals and send them to the intracellular environment (Tan et al., 2019). In this way, they respond to environmental stress. Depending upon their extracellular regions, RLKs are classified into various types, such as leucine-rich repeat RLKs, S-domain RLKs, cysteine-rich RLKs, and wall-associated RLKs (X. Zhao et al., 2022).

Cysteine-rich RLKs (CRKs) form a major group of RLKs having more than 40 members in *Arabidopsis thaliana*. Two copies of the domain of unknown function 26 (DUF26; PFAM domain PF01657), which contains four conserved cysteines, make up the protein’s extracellular region. These three cysteines together make up the motif C-8X-C-2X-C, which may play a role in the production of disulfide bridges that might be used to regulate thiol redox (Chen et al., 2004; Shiu and Bleecker, 2003; Wrzaczek et al., 2010). CRKs are linked to reactive oxygen species (ROS) signaling and cell death. In *A. thaliana*, the kinase CRK2 plays a crucial role in innate immunity and growth of plants by forming a complex with an NADPH oxidase respiratory burst oxidase homolog D (RBOHD), in response for extracellular ROS production. CRK2’s activity is vital for ROS bursts in response to elicitors, impacting defense against pathogens. Phosphorylation of RBOHD’s C-terminal enhances ROS production, suggesting an evolutionarily conserved mechanism, highlighting CRK2’s role in regulating ROS in response to microbial patterns (Kimura et al., 2020). In CRKs, oxidative stress, pathogen attack, and salicylic acid induction are the major causes of transcriptional induction (Chen et al., 2003). Several *Arabidopsis CRK* members regulate the defense response against pathogens and cause cell death in leaves. Moreover, over-expression of *CRK5* causes increased resistance toward a virulent pathogen *Pseudomonas syringae*. Similarly, over-expression of *CRK4/5/19* and 20 by a chemically inducible promoter causes cell death. Genetic analyses have suggested the involvement of *CRK5* in the regulation of cell death independent of SA. However, increased resistance to *Pseudomonas* due to the over-expression of *CRK13* requires increased levels of SA (Acharya et al., 2007).

CRKs have been identified in several plant species, and a number of them have been elucidated for their biological functions. These reports have demonstrated that they are mainly involved in hormonal signaling pathways, tolerance to environmental stresses, and plant growth. In *Arabidopsis*, higher expression of *CRK1*, also known as *AtCBK3*, results in an increased thermotolerance (Wei et al., 2019). Moreover, previous studies have shown the association between *CRK3* and cytosolic glutamine synthetase (GLN1), which mobilized nitrogen during leaf senescence, while *CRK1* and *CRK5* conferred drought stress tolerance by negatively regulating ABA signaling (Li et al., 2006). Furthermore, *AtCRK6*,*7* and *HvCRK1* from *Hordeum vulgare* were found to enhance a regulatory response against the pathogen, powdery mildew (Rayapuram et al., 2012; Bourdais et al., 2015). The *TaCRK1* gene in wheat showed an upregulated expression in response to a pathogen, *Rhizoctonia cerealis* (Saintenac et al., 2021). This reveals the involvement as well as the importance of *CRK* genes in physiological processes during plant development (Sarwar et al., 2023).

*Arachis hypogaea* L., also commonly known as peanut and groundnut, is an oilseed and grain legume which is extensively cultivated in the tropical and subtropical regions, with a yearly production of nearly 46 million tons. The genus *Arachis* is prevalent in South America and comprises mostly the diploid species (2n = 2x = 20), whereas *A. hypogaea* is an allotetraploid (AABB-type genome; 2n = 4x = 40) resulting from the hybridization occurrence between two diploid species, followed by polyploidization (Bertioli et al., 2016). Homologous A and B genomes *Arachis duranensis* (AA, 2n = 20) and *Arachis ipaensis* (BB, 2n = 20) contributed to the hybridization (Zhang et al., 2017). Peanut is rich in oil (40%–60%), carbohydrates, protein (10%–20%), minerals, vitamins, monosaturated fatty acids, and antioxidants. India is the largest consumer of edible oil worldwide, and it also consumes the most peanut oil (Patel et al., 2022). Semi-arid tropics (SAT) including Asia, Africa, and South and North America account for approximately 60% of peanut production worldwide. These areas have predominant extremes of drought, salinity, and temperature. These conditions such as salt and drought stress significantly affect the growth and productivity of plants. Estimates have shown that drought stress causes the loss of approximately 6 million tons of peanuts, which is worth about 250 USD. Similarly, soil salinity halts plant growth by reducing the mineral uptake by the plant (Banavath et al., 2018). Peanut production is greatly affected by heat, drought, and salt stresses as it grows in tropical and subtropical regions. Since CRKs are involved in the defense mechanism of plants against environmental stresses, the identification of peanut CRKs can help understand their interaction mechanisms. The availability of *A. hypogaea*, *A. duranensis*, and *A. ipaensis* genomes has facilitated genome-wide identification as well as the characterization of CRKs. The aim of studying and characterizing the inter- and intra-species diversity led to the pangenome-wide analysis of these three peanut genomes. A comprehensive structural evaluation, including gene structure, motif analysis, phylogenetics, chromosomal distribution, and gene enrichment, has been performed. In addition, the differential expression of the identified members under drought and salt stress has also been carried out to find multi-stress-related genes. Furthermore, their involvement in multi-stress responsiveness is also validated through a machine learning classifier algorithm. Hence, this study will broaden our knowledge of the CRK gene family in peanut, elucidating their contribution to conferring resistance against various environmental stresses, and also will serve as valuable insights for future researchers.

## 2 Materials and methods

### 2.1 Identification and characterization of the CRK gene family in *A. hypogaea*, *A. duranensis*, and *A. ipaensis*

The 44 *A. thaliana* CRK protein sequences were retrieved from the NCBI protein database (https://www.ncbi.nlm.nih.gov/protein/). The protein sequence FASTA files of *A. hypogaea*, *A. ipaensis,* and *A. duranensis* were downloaded from the NCBI. NCBI command-line tool, BLAST+, was used to create a local database of these files. A BLASTp search was performed against these protein sequence databases, using *Arabidopsis* CRK protein sequences as queries. The resulting hits were further refined by removing duplicates and isoforms.

Furthermore, the identified proteins were searched to confirm the presence of the stress antifungal/DUF26 (PF01657) and protein kinase (PF00069 and PF07714) domains. For this purpose, NCBI conserved domain database (CDD) (https://www.ncbi.nlm.nih.gov/Structure/cdd/wrpsb.cgi) (Marchler-Bauer et al., 2015), Simple Modular Architecture Research Tool (SMART) (http://smart.embl-heidelberg.de/) (Schultz et al., 2000), and InterPro (https://www.ebi.ac.uk/interpro/) (Hunter et al., 2009) database were utilized. The proteins having no characteristic conserved domains were excluded from further analysis. Furthermore, TBtools (Chen et al., 2018) was used to construct the domain architecture.

Information on the various physicochemical properties [molecular weight, their isoelectric point (pI), instability index (II), aliphatic index (AI), and the grand average of hydropathicity (GRAVY)] was predicted by using the ExPASy ProtParam tool (https://web.expasy.org/protparam/) (Gasteiger et al., 2005). Subcellular localization for each three peanut CRKs was predicted using an online WoLF PSORT tool (https://wolfpsort.hgc.jp/) (Horton et al., 2007).

### 2.2 Phylogenetic, gene structure, and conserved motif analysis of AhCRKs

A phylogenetic tree was constructed to evaluate the evolutionary links among CRK proteins. A multiple sequence alignment of 71 *A. hypogaea* (AhCRKs) 36 *A. duranensis* (AdCRKs), 44 *A. ipaensis* (AiCRKs), 44 *A. thaliana* (AtCRKs) (K. Chen et al., 2004), 37 *H. vulgare* (HvCRKs) (Rayapuram et al., 2012), 36 *Oryza* *sativa* (*OsCRKs*) (Shumayla et al., 2019) and 46 *Proteus* *vulgaris* (*PvCRKs*) (Quezada et al., 2019) was done using ClustalW (Zameer et al., 2021). A phylogenetic tree was constructed using the IQ-TREE web server (http://iqtree.cibiv.univie.ac.at/) (Trifinopoulos et al., 2016). Using the maximum likelihood (ML) approach and 1,000 bootstrapping replicates, the reliability of the built-in tree was confirmed. Further editing of the tree was done using the Interactive Tree of Life (iTOL) (https://itol.embl.de/) (Letunic and Peer, 2021).

The conserved motif among the members of the CRK family of each three *Arachis* species was searched using the Multiple Expectation Maximization for Motif Elicitation (https://meme-suite.org/meme/tools/meme) tool (Bailey et al., 2015). A maximum of 20 conserved motifs were analyzed. The GFF files of each *Arachis* species were used to analyze the intron–exon pattern. Both the motifs and gene structures were visualized using TBtools (Zia et al., 2022).

### 2.3 Chromosomal location, Ka/Ks, and gene duplication analysis

The chromosomal location information for *CRK* genes from each of the three *Arachis* spp*.* was determined from the NCBI genomic database. The distribution of *CRK* genes from three *Arachis* species across their chromosomes was analyzed and visualized using TBtools. Duplicated pairs of *AhCRK* genes were identified using NCBI nucleotide BLAST (https://blast.ncbi.nlm.nih.gov/Blast.cgi?PROGRAM=blastn&PAGE_TYPE=BlastSearch&LINK_LOC=blasthome) (Zia et al., 2022), based on the coverage of the aligned sequences which should be ≥ 70%. The Ka/Ks ratios (the rate of non-synonymous/synonymous substitution) for the duplicated gene pairs were also calculated using DnaSP v.6 software (Rozas et al., 2017). This was done to assess the molecular evolutionary rates of each gene pair. Moreover, the time of divergence for these gene pairs was calculated using the formula “t = Ks/2λ,” with a λ value of 1.5 × 10^−8^ for dicots, substitutions/synonymous site, and year representing the neutral substitution (Zameer et al., 2022). This was shown a million years ago (Mya). The linkage between chromosomes and duplicated pairs was shown using the Advanced Circos program of TBtools.

### 2.4 Protein–protein interaction, Gene Ontology enrichment, and miRNA prediction

Amino acid sequences of AhCRKs were subjected to the STRING database (https://string-db.org/) (von Mering et al., 2003) to analyze the interactions among peanut CRKs and other proteins. The top 10 interactions were set to be predicted, and the threshold level was kept medium (0.4). The interaction network was visualized using Cytoscape software (Shannon et al., 2003). The components considered for GO enrichment were biological processes (BPs), cellular components (CCs), and molecular functions (MFs), and these were predicted using the DAVID database (https://david.ncifcrf.gov/home.jsp) (Dennis et al., 2003). Moreover, KEGG pathways were also analyzed using the same database. The miRNAsong database (miRNAsong - A tool for microRNA sponge sequence generation and testing (muni.cz)) (Barta, Peskova, and Hampl, 2016) was used to identify the *A. hypogaea* miRNAs. Furthermore, the psRNATarget database [psRNATarget: A Plant Small RNA Target Analysis Server (2017 Update) ( zhaolab.org)] (Dai and Patrick, 2011) was used to identify the putative miRNAs targeting the *AhCRK* genes using the coding sequences of *AhCRKs* as target sequences.

### 2.5 *Cis*-regulatory elements and expression profiling of *AhCRKs* in abiotic stresses

For identification of *cis*-regulatory elements, 2 kb sequences upstream of the translation start site of *CRK* genes from each of the three *Arachis* species were extracted and scanned using the PlantCARE online tool (http://bioinformatics.psb.ugent.be/webtools/plantcare/html/) (Rombauts et al., 1999). Finally, the diagram was illustrated using TBtools software.

The expression levels of all *AhCRK* genes in peanut leaves under drought and salt stress were evaluated using transcriptome datasets available at the NCBI Sequence Read Archive (SRA) database (https://www.ncbi.nlm.nih.gov/sra) (BioProjects; PRJNA706902: drought stress and PRJNA603232: salt stress). The genome and annotation (GFF) files were downloaded from the genome-NCBI database (https://www.ncbi.nlm.nih.gov/genome/). Indexes of the *A. hypogaea* genome were built by using Bowtie2 (Colling et al., 2013) and the clean paired-clean reads were then mapped to the genome. Furthermore, the gene level counts from RNA-seq providing the expression level of the genes were obtained by using featureCounts (Liao et al., 2014). The count values for each condition were used to generate a heatmap. The logarithmic transformed values were used to show the differential expression of genes.

### 2.6 Evaluation of multi-stress-related *AhCRK* genes using machine learning

DESeq2 (Love et al., 2014) was applied to both drought and salt samples to identify the genes which were significantly expressed. The genes were further divided based on their statistical significance depending on whether their *p*-value is less than 1 and the log_2_ fold-change values (log_2_ fold value >0.5 for upregulation and log_2_ fold value <0.5 for downregulation). The *AhCRK* genes common to both datasets were collected. Furthermore, to access the credibility of these genes, the classification algorithm—random forest (RF) was implied in R. Assessing the performance of a model is usually subjective, which involves comparing the model’s predictions to the known values of the dependent variable in a specific dataset. DESeq normalization was applied to the salts’ count datasets for the RF classifiers, in which the genes common to both stresses were incorporated. The performance of the RF classifier was evaluated using matrices including the accuracy, the area under the receiver operating characteristic curve (AUC), specificity, and sensitivity of the data with common multi-stress-related genes.

### 2.7 3D structure prediction of AhCRK proteins

**FIGURE 1:**
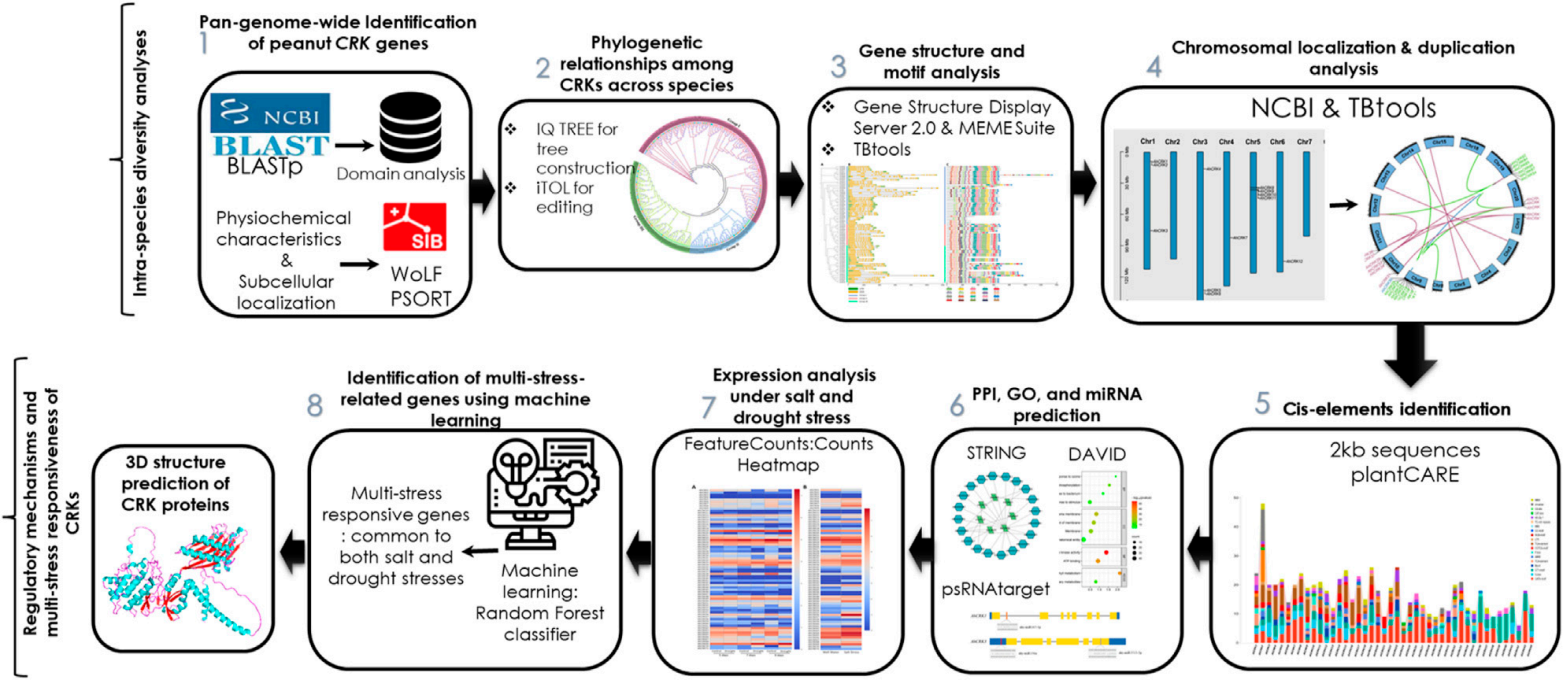
Workflow of the pangenome-wide identification of *CRK* genes in peanut, their structural and functional analysis, expression profiling, and multi-stress responsiveness.

The three-dimensional (3D) structure of a protein is necessary for its proper functioning. Based on the expression analysis, the 3D structures of three AhCRK proteins were predicted using AlphaFold2 (https://colab.research.google.com/github/sokrypton/ColabFold/blob/main/AlphaFold2.ipynb) (Jumper et al., 2021). The predicted structures were validated using the SAVES (https://saves.mbi.ucla.edu) server (Elshemey et al., 2010) and MolProbity (http://molprobity.biochem.duke.edu/) (Davis et al., 2007). PyMOL (Alexander et al., 2011) was used to visualize these structures. The overview of the current study is shown in Figure 1.

## 3 Results

### 3.1 Identification of *CRK* genes in three *Arachis* species

A total of 71, 36, and 44 genes were identified in *AhCRKs*, *AdCRKs*, and *AiCRKs* (Supplementary Tables S1–S3). The protein domain analysis of these identified CRKs in three *Arachis* species confirmed the presence of the conserved stress-antifungal/DUF26 (PFAM PF01657) and Pkinases (PFAM domains PF00069 and PF07714) domains. All the proteins from these three species contained two to four PF01657 (DUF26; salt stress response/antifungal domain). For Pkinase domains, some of the genes contained PF00069 (protein kinase domain) and others had PF07714 (protein tyrosine kinase domain) conserved in them (Supplementary Tables S4). Furthermore, all the genes were named in chronological order based on their position on chromosomes.

**FIGURE 2:**
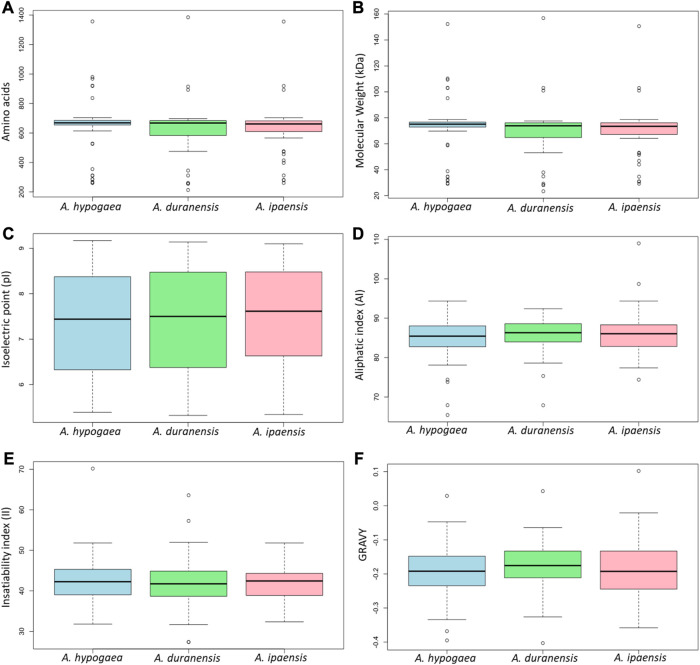
Box plots showing the physiochemical characteristics of three *Arachis* species: **(A)** amino acid residues/protein length, **(B)** their molecular weight, **(C)** their isoelectric point, **(D)** aliphatic index, **(E)** insatiability index, and **(F)** the grand average of hydropathicity.

The physicochemical properties of 71 identified *Arachis* CRK proteins were analyzed. There were no substantial differences in their protein length/amino acid residues, molecular weights, isoelectric point, instability index, aliphatic index, and GRAVY values among the three species. In all the three species, most of these proteins have pI values ranging between 5 and 9, indicating their acidic as well as basic behavior. The II values of most of these proteins showed that they will be unstable in the test tube. Almost all of these proteins had an AI greater than 70, which indicates that these proteins are thermally stable, and negative GRAVY values indicate that these proteins are hydrophilic (Figure 2). The determination of subcellular localization of AhCRK proteins will help understand their molecular functions. Most of the AhCRKs were localized in the plasma membrane, which was followed by the extracellular membrane, and chloroplast. In *A. duranensis*, AdCRKs were localized in the plasma membrane, chloroplast, and extracellular space. In *A. ipaensis*, these proteins were also localized in the plasma membrane, extracellular membrane, and chloroplast (Supplementary Tables S1–S3).

### 3.2 Phylogenetic relations of *Arachis* CRK proteins

**FIGURE 3:**
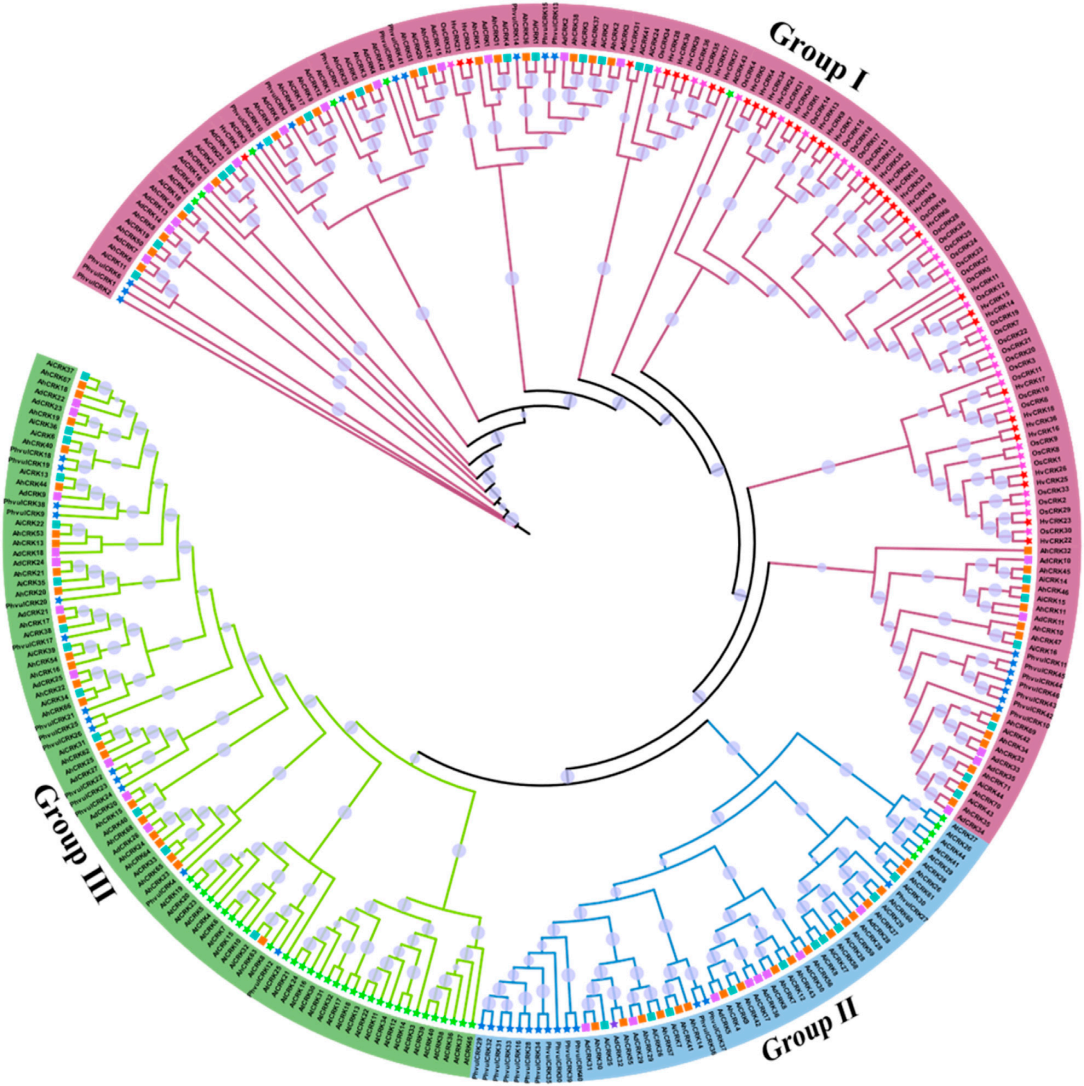
Phylogenetic tree of the CRK protein sequences from seven different plant species including three *Arachis* species, generated using the maximum likelihood method. Different groups are represented by specific clade and branch colors.

The identified protein sequences from *A. hypogaea, A. duranensis*, *A. ipaensis*, *A. thaliana*, *O. sativa*, *H. vulgare*, and *P*. *vulgaris* were used in the construction of the phylogenetic tree to study intra- and inter-species diversity. According to the phylogenetic tree, these protein sequences were classified into three groups, and each group contained a different number of members from each species (Figure 3). Group I had the maximum number of members present in it (166 members). Members from all seven species were present in this group, showing the shared homology among them. This group contained 30 members from *A. hypogaea*, 21 members from *A. ipaensis*, 16 members from *A. duranensis*, six members from *A. thaliana*, and 36 members from *O. sativa*.

Group II, the smallest group with 57 members, has the following members: 18 *A. hypogaea*, 10 *A. ipaensis*, nine *A. duranensis*, six *A. thaliana*, and no members from *O. sativa*. Group III has 92 members: 23 *A. hypogaea*, 13 *A. ipaensis*, 10 *A. duranensis*, and 32 *A. thaliana*. This group does not have any members from *Oryza sativa* as well. The results suggested the close evolutionary relationships across species, which indicates structural and functional conservations as well. Members of all three *Arachis* species were clustered in all three groups, indicating their intraspecies conservation. The presence of members from other species in groups also indicates the orthologous relationships among species.

### 3.3 Gene structure and the conserved motif analysis

Gene length in *A. hypogaea* varied from 938 bp (*AhCRK42*) to 26,515 bp (*AhCRK32*). Exon and intron positions were associated and compared to increase our insights into the structural diversity among the CRK members of *A. hypogaea,* as well as intraspecies diversity among the members from three *Arachis* species. For *A. hypogaea,* the intron number varied from 1 (*AhCRK63*) to 16 (*AhCRK35*). Moreover, some members including *AhCRK4*, *AhCRK42,* and *AhCRK65* contained no intronic regions. Members from each group contained a similar number of introns and exons.

**FIGURE 4:**
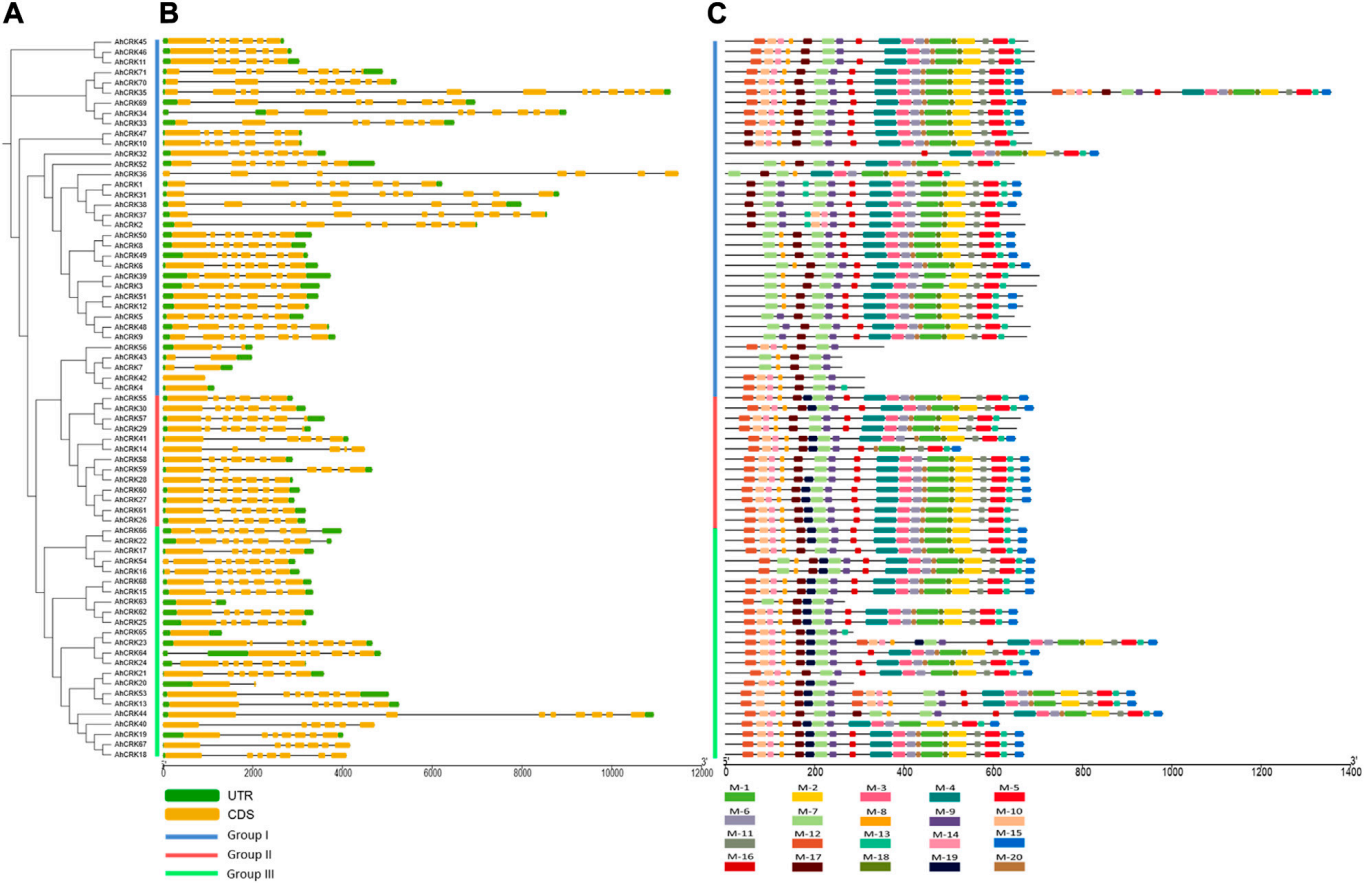
**(A)** Phylogenetic tree of AhCRKs, **(B)** structural features showing exon–intron organization, and **(C)** a conserved motif pattern of 71 AhCRK proteins.

In *A. duranensis*, the gene length ranged from 855 bp (*AdCRK17*) to 19,199 bp (*AdCRK9*). The exon–intron pattern was also conserved among members of the same group. Group I contained zero to seven introns, whereas members of Group II contained a maximum of seven introns, and the members of Group III had introns ranging from five to eleven. For *A. ipaensis*, the observed gene length varied from 873 bp (*AiCRK8*) to 10,977 bp (*AiCRK1*). The members from the same group showed conservation among their gene structures, while the overall number of introns ranged from 0 (*AiCRK8* and *AiCRK12*) to 10 (*AiCRK43*). This variation in the number of introns and exons might have resulted in varied gene lengths among the members from the same group, as well as intraspecies (Figure 4; Supplementary Figures S1, 2).

The occurrence of 20 conserved motifs was analyzed among *AhCRK*, *AdCRK*, and *AiCRK* proteins. For *AhCRKs*, almost 18 motifs were conserved among the maximum members. Some members showed a fewer number of conserved motifs, whereas members of group 2 had a higher conservation of motif patterns. In *A. duranensis*, almost 16 motifs were conserved among most members. Members of Group I had slight variations in their conservation pattern, while those of Group II and Group III showed significant conservation of motif patterns. Members of AiCRKs showed the greatest conservation of motifs among all the members and groups with very few members having a fewer number of motifs conserved in them.

### 3.4 Chromosomal mapping of *CRKs* and their duplication analysis

**FIGURE 5:**
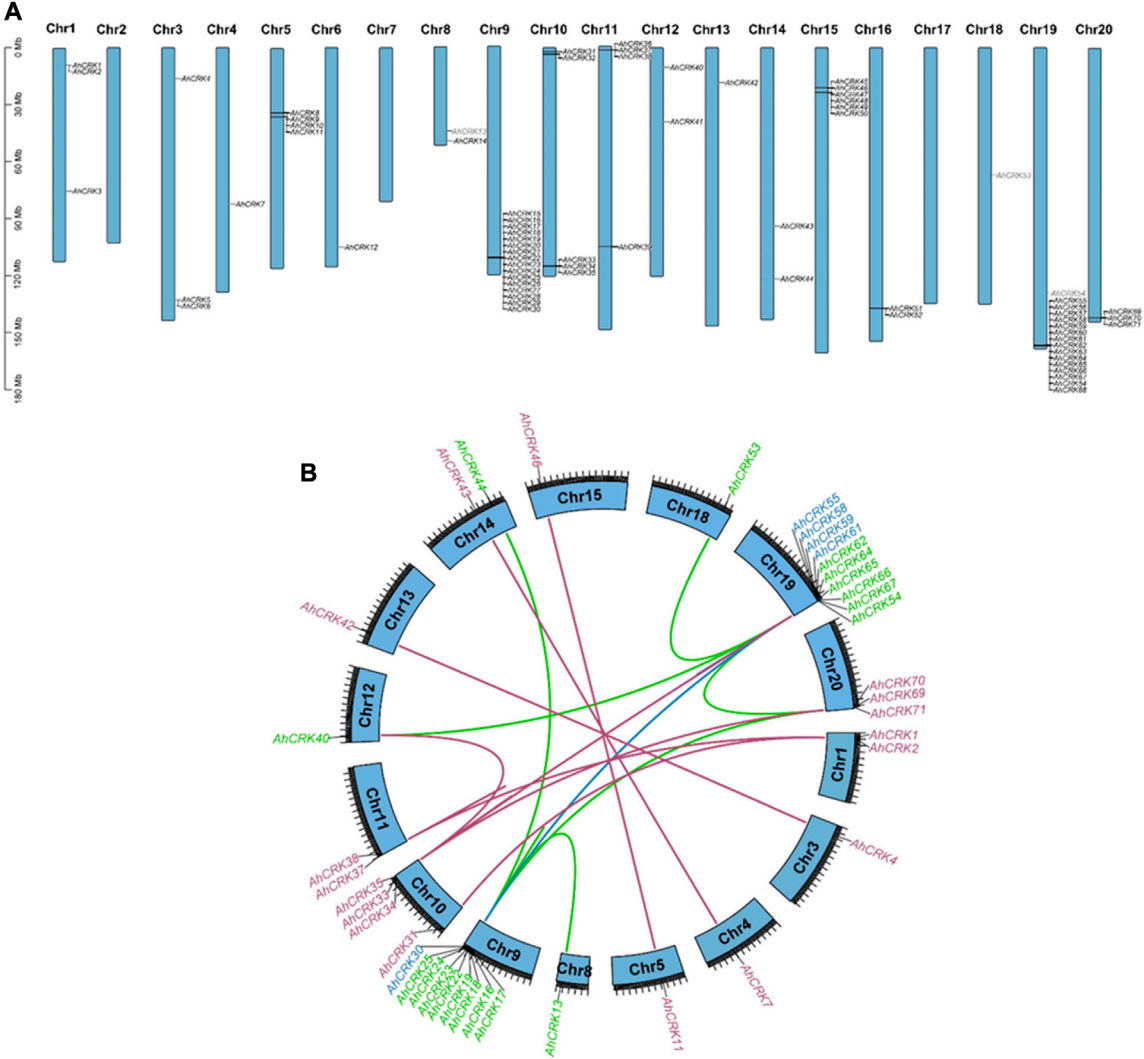
**(A)** Chromosomal mapping of *AhCRK* genes; **(B)** segmental and tandem duplications among the *AhCRK* members. Gene label colors specify the group they belong to.

To evaluate the pattern of genomic distribution of *AhCRK*, *AdCRK*, and *AiCRK* genes, their chromosomal gene location was identified. Furthermore, duplication events of these genes were analyzed using syntenic analysis. These analyses showed that the *AhCRK* genes were found to be randomly distributed on 17 out of 20 *A. hypogaea* chromosomes. Peanut Chr9 and Chr19 had the highest number of genes (16 members) mapped on them. Chr2, Ch7, and Chr17 had no CRK genes present in them (Figure 5). In the *A. duranensis* genome, eight chromosomes had *AdCRK* genes mapped on them, and Chr2 and Chr7 with no CRK genes mapped onto them. Consistent with the mapping pattern observed in *A. hypogaea*, Chr9 of the *A. duranensis* genome had the highest number of genes clustered on it (14 *AdCRKs*). Only one gene *AdCRK36* was present on the scaffold. *A. ipaensis* also followed a similar mapping pattern, with genes mapped on every chromosome except Chr7 and the highest number of *AiCRKs* being clustered on Chr9 (18 genes). These results show the conservation in gene position patterns on chromosomes **(** Supplementary Figures S3, 4 **)**.

Gene duplication events were also observed among *AhCRK*, *AdCRK*, and *AiCRK* genes, and a total of 41, 14, and 20 duplicated gene pairs were found in these members, respectively. In *A. hypogaea*, 10 pairs of tandem and 31 pairs of segmental duplication were observed. In *A. duranensis*, 11 pairs of tandemly duplicated genes were found, with three pairs being segmentally duplicated. Similarly, in *A. ipaensis*, 13 pairs of genes resulting from tandem duplication were analyzed with seven gene pairs being segmentally duplicated. These observations are consistent with their genomic composition; for instance, *A. hypogaea* have double the size of the genome as that of *A. duranensis* and *A. ipaensis*¸ indicating that the *AhCRK* genes are dispersed on different chromosomes. Segmental supplication is the duplication of genes from one chromosome onto another; thus, the presence of more segmental duplication pairs in *A. hypogaea* makes sense. To analyze the evolutionary constraints of the repeated *Arachis CRK* genes, the Ka, Ks, and the Ka/Ks ratios of all para-homologous gene pairs were then calculated. In *A. hypogaea*, almost half of the gene pairs had their Ka/Ks values ranging from 0.16 to 0.99, which were smaller than 1.0, indicating significant purification selection pressure had been applied to these gene pairs. The rest of the half pairs having Ka/Ks values more than 1.0 indicated that positive selection also occurred. As a result, the divergence time of 41 duplicated pairs was between 0.67 Mya and 117.42 Mya. In *A. duranensis*, three gene pairs exhibited positive selection (Ka/Ks values >1), while the rest of the duplicated pairs showed negative selection (Ka/Ks values <1). Furthermore their divergence time ranged from 1.653 to 121.91 Mya. For *A. ipaensis*, eight duplicated gene pairs showed positive selection, and the rest of the 12 pairs showed purifying selection, with their divergence time ranging from 0.84 to 129.4 Mya (Table 1; Supplementary Tables S5, 6).

**TABLE 1:** Duplication data of *AhCRK* genes, synonymous and non-synonymous mutations, duplication time, and type of duplication between the genes.

Gene1	Gene2	Ka	Ks	Ka/Ks	Time (Mya)	Duplication type
*AhCRK1*	*AhCRK31*	0.0347	0.0214	1.621495327	7.45	Segmental
*AhCRK2*	*AhCRK37*	3.7496	2.3624	1.587199458	78.74666667	Segmental
*AhCRK4*	*AhCRK42*	2.6498	2.6665	0.993737109	88.88333333	Segmental
*AhCRK7*	*AhCRK43*	0.0068	0.0432	0.157407407	1.44	Segmental
*AhCRK11*	*AhCRK46*	0.0193	0.0269	0.717472119	0.896666667	Segmental
*AhCRK13*	*AhCRK16*	2.5913	2.2459	1.153791353	74.86333333	Segmental
*AhCRK13*	*AhCRK53*	3.2281	2.8876	1.117917994	96.25333333	Segmental
*AhCRK16*	*AhCRK17*	1.0659	0.824	1.293567961	27.46666667	Tandem
*AhCRK16*	*AhCRK23*	1.0599	1.2966	0.817445627	43.22	Tandem
*AhCRK16*	*AhCRK54*	4.5769	2.1359	2.142843766	71.19666667	Segmental
*AhCRK17*	*AhCRK23*	1.159	1.2621	0.918310752	42.07	Tandem
*AhCRK18*	*AhCRK44*	2.0386	1.8623	1.094667884	62.07666667	Segmental
*AhCRK18*	*AhCRK62*	1.1394	1.4638	0.778385025	48.79333333	Segmental
*AhCRK18*	*AhCRK66*	0.913	0.7892	1.156867714	26.30666667	Segmental
*AhCRK19*	*AhCRK62*	1.1711	1.4638	0.800040989	48.79333333	Segmental
*AhCRK19*	*AhCRK66*	0.8902	0.7892	1.127977699	26.30666667	Segmental
*AhCRK22*	*AhCRK66*	4.4435	1.8972	2.342135779	63.24	Segmental
*AhCRK22*	*AhCRK67*	0.3291	1.3001	0.253134374	43.33666667	Segmental
*AhCRK23*	*AhCRK24*	3.5095	1.3222	2.654288307	44.07333333	Tandem
*AhCRK23*	*AhCRK64*	0.0319	0.0301	1.059800664	1.003333333	Segmental
*AhCRK23*	*AhCRK65*	0.021	0.0201	1.044776119	0.67	Segmental
*AhCRK23*	*AhCRK69*	2.872	0.9522	3.016173073	31.74	Segmental
*AhCRK24*	*AhCRK65*	4.5046	1.3529	3.329588292	45.09666667	Segmental
*AhCRK25*	*AhCRK54*	0.6632	1.0895	0.608719596	36.31666667	Segmental
*AhCRK30*	*AhCRK55*	0.2169	0.1422	1.525316	4.74	Segmental
*AhCRK30*	*AhCRK61*	0.4717	0.4178	1.129009	13.92667	Segmental
*AhCRK33*	*AhCRK70*	0.0852	0.3206	0.265752	10.68667	Segmental
*AhCRK33*	*AhCRK71*	0.0632	0.3849	0.164198	12.83	Segmental
*AhCRK34*	*AhCRK35*	2.0286	2.2588	0.898087	75.29333	Tandem
*AhCRK34*	*AhCRK69*	0.0512	0.0353	1.450425	1.176667	Segmental
*AhCRK35*	*AhCRK40*	2.4207	2.861	0.846103	95.36667	Segmental
*AhCRK35*	*AhCRK67*	2.0409	1.6075	1.269611	53.58333	Segmental
*AhCRK35*	*AhCRK69*	1.9226	3.5227	0.545775	117.4233	Segmental
*AhCRK37*	*AhCRK38*	0.4334	0.1753	2.472333	5.843333	Tandem
*AhCRK40*	*AhCRK67*	0.1792	1.1041	0.162304	36.80333	Segmental
*AhCRK53*	*AhCRK66*	1.2481	0.8689	1.436414	28.96333	Segmental
*AhCRK55*	*AhCRK61*	0.5376	0.3274	1.642028	10.91333	Tandem
*AhCRK58*	*AhCRK59*	2.0327	1.6183	1.256071	53.94333	Tandem
*AhCRK64*	*AhCRK65*	0.014	0.0404	0.346535	1.346667	Tandem
*AhCRK66*	*AhCRK67*	3.0929	2.4108	1.282935	80.36	Tandem
*AhCRK67*	*AhCRK71*	0.5858	1.3786	0.424924	45.95333	Segmental

Mya*: million years ago.

### 3.5 *Cis*-regulatory element analysis of *Arachis* spp*.*

**FIGURE 6:**
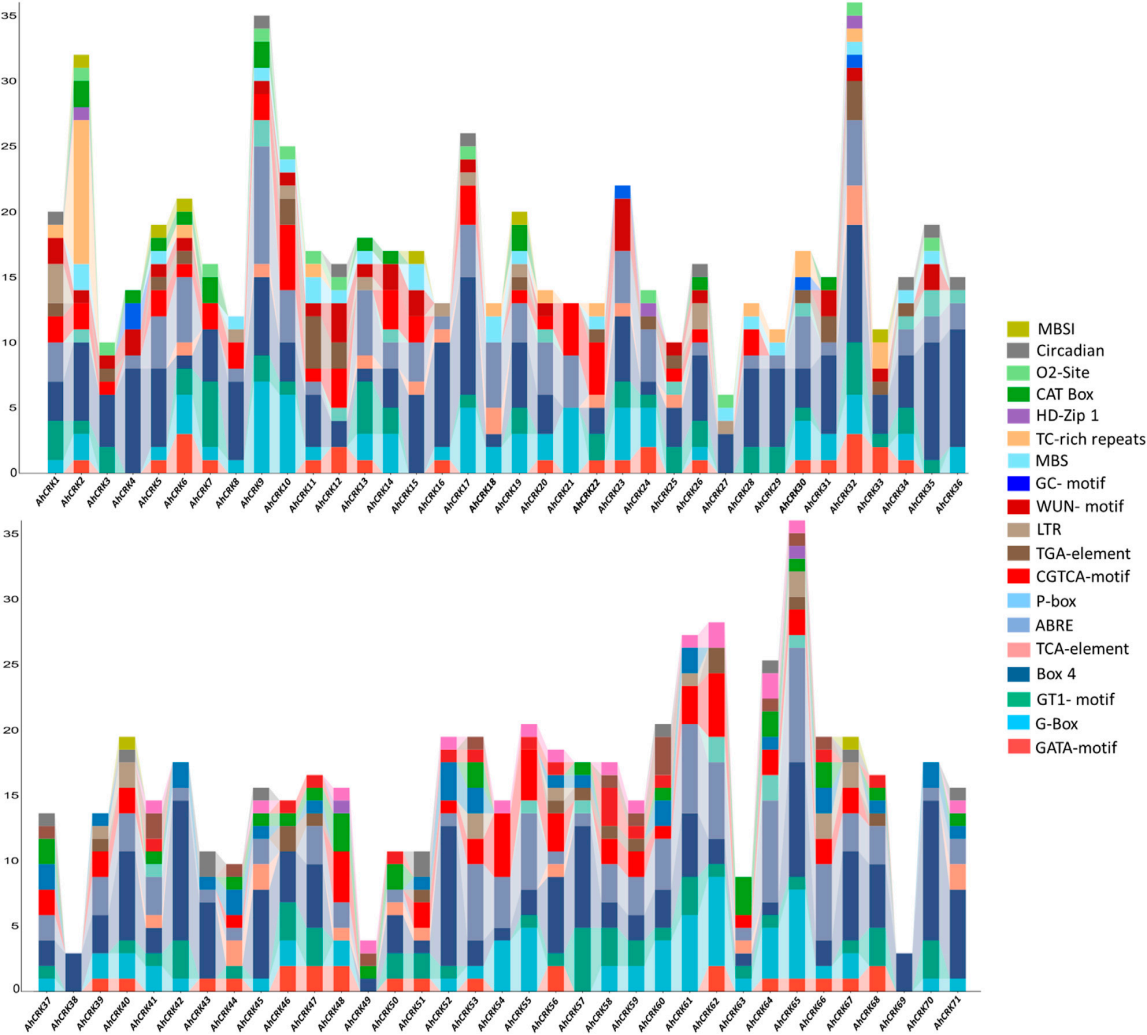
*Cis*-regulatory elements in the upstream promoter regions of the *AhCRK* genes. Each bar is representing the specific elements present in the particular gene.

To get better insights and understanding regarding the functional roles of *Arachis CRK* genes, their upstream promoter regions were analyzed to predict the *cis*-acting elements present in them. Several development-related (MBSI, Circadian, O_2_-site, CAT-Box, and HD-Zip 1), stress-related (TC-rich repeats, MBS, LTR, GC-motif, and WUN-motif), hormone-related (P-box, TGA-element, CGTCA-motif, ABRE, and TCA-element), and light-related (GATA-motif, Box 4, GT1-motif, and G-Box) *cis*-regulatory elements were identified in each *Arachis* CRK members’ promoters (Figure 6).

In *A. hypogaea*, all four types of elements were observed in each *AhCRK* members’ promoters’ site, except for *AhCRK38* and *AhCRK69* which contained only one light-related element (Box 4). All these elements corresponded to gibberellin, auxin, abscisic acid, and MeJA responsiveness; endosperm and meristem expression; low-temperature responsiveness; and zein metabolism regulation. Each of the *A. duranensis CRKs* contain all four kinds of *cis*-regulatory elements. Members of each *A. ipaensis CRKs* contain all four types of *cis*-acting elements with a large number of light-related elements (GATA-motif). Moreover, *AiCRK38–40* and *AiCRK42* contained only light-responsive elements (Supplementary Figures S5, 6). All these results demonstrate not only the conservation of elements but also their potential involvement in growth, developmental, hormonal, and stress-related processes leading to their functional roles in the tolerance of environmental stresses.

### 3.6 Prediction of miRNAs, protein–protein interaction network, and Gene Ontology enrichment analysis

**FIGURE 7:**
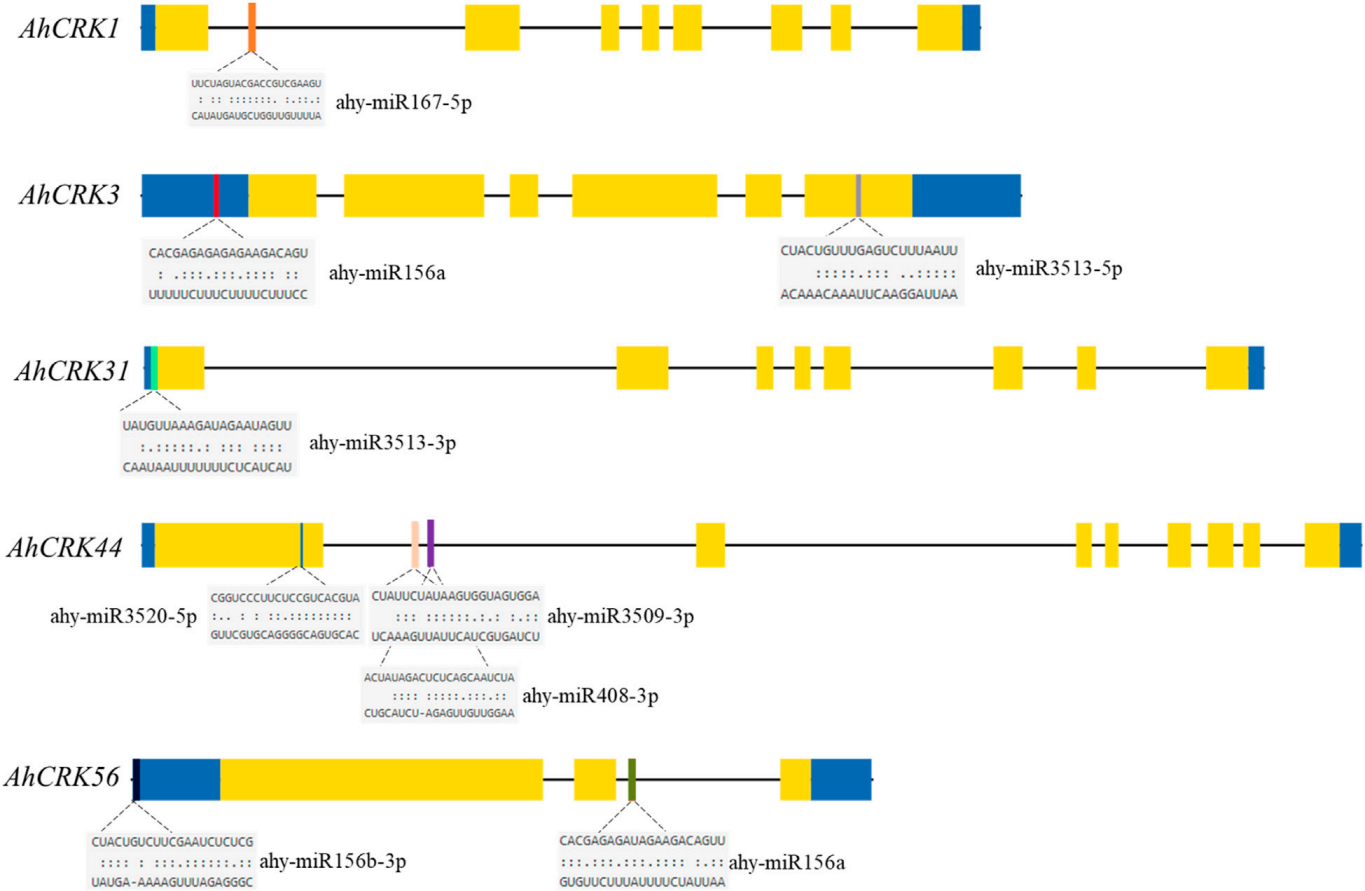
Figure shows the predicted miRNAs potentially targeting the *AhCRKs* and the target sites.

Several studies in recent years have unveiled the regulatory roles of miRNAs in the transcription and expression of genes under various developmental and stress-related conditions. Therefore, the miRNAs targeting the *AhCRK* genes were predicted to get insights into the miRNA-mediated post-transcriptional regulation of these genes. A total of 34 *AhCRKs* were targeted by miRNAs from 12 different families (Figure 7; Supplementary Table S7). Members of the miR156 family targeted *AhCRK3*, *AhCRK20*, *AhCRK49*, *AhCRK55*, and *AhCRK56*. miRNAs from the miR160 family targeted *AhCRK32*. Similarly, miRNAs of the miR167 family targeted *AhCRK1* and *AhCRK38*. Further studies are required to determine the biological roles of these peanut miRNAs and their involvement in gene expression mechanisms.

**FIGURE 8:**
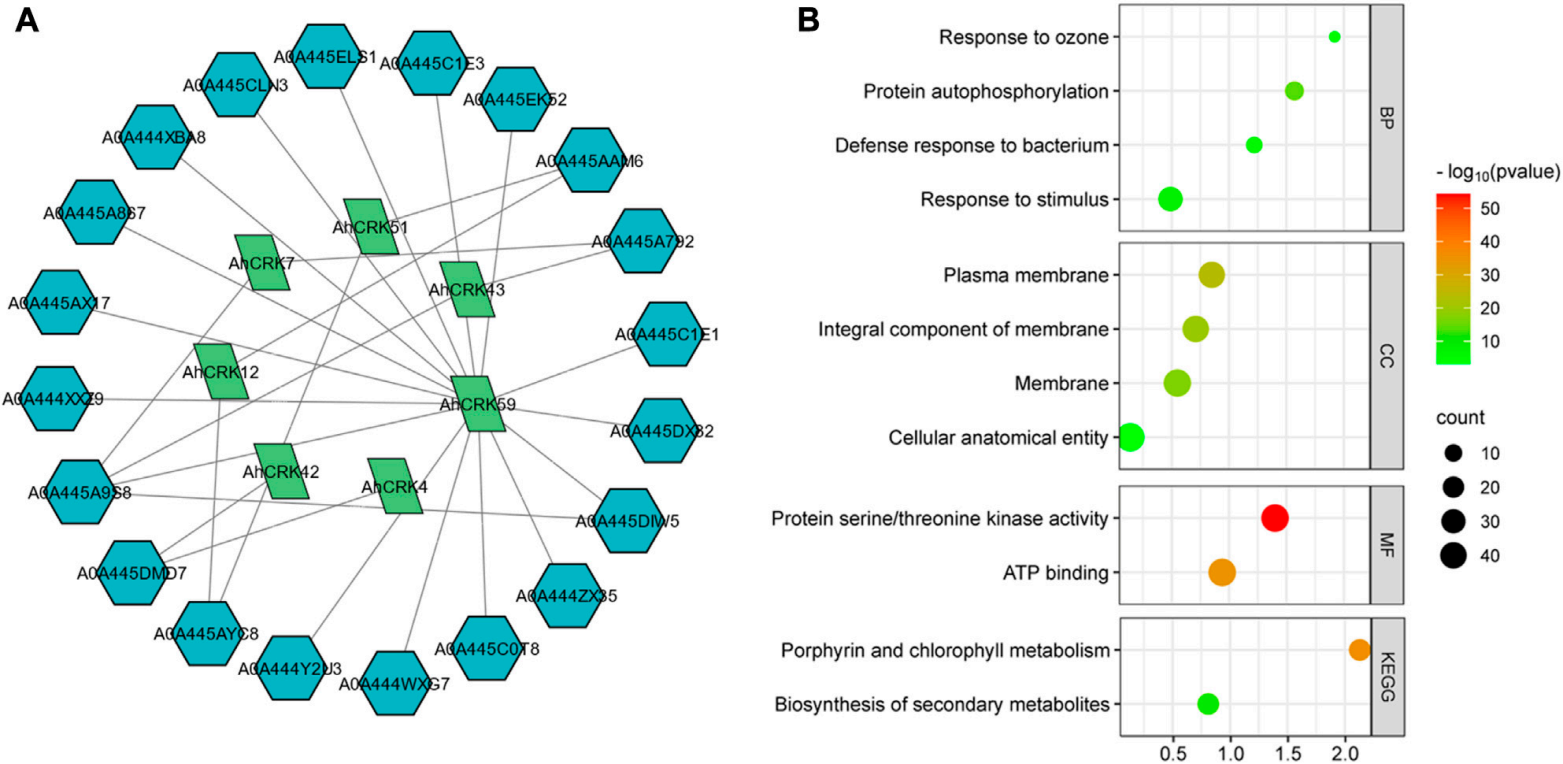
**(A)** Network showing the interactions among AhCRK protein members and other related proteins. The green nodes are AhCRKs, and the blue nodes are other interacting proteins. **(B)** GO enrichment bubble plot representing the biological processes, their cellular components, potential molecular functions, and GO and KEGG pathways in which AhCRK proteins are potentially involved.

A PPI network of the *Arachis* CRK proteins was also generated to understand the functional relativity among them (Figure 8A). The *Arachis* CRKs interacted with each other and other proteins showing connectivity as well as their functional relativity. More specifically, AhCRK59, AhCRK57, and AhCRK23 interacted with most of the other related proteins. These relative proteins were found to be mostly involved in defense and immunity response, the signaling pathway associated with transmembrane receptor protein tyrosine kinase, and the signal transduction which suggests the potential role of AhCRKs in related pathways and mechanisms.

GO enrichment analysis was then performed to further determine the dynamic roles of *CRKs* at the molecular level. Based on this GO analysis, *AhCRK* genes were classified into three different major categories: biological processes (BP), cellular components (CC), and molecular functions (MF). Biological processes in which these proteins were found to be involved included responses to stimulus and defense responses. Almost all of the proteins were found to have membranes as their cellular component. Similarly, their molecular functions included kinase activity and ATP binding. Their related KEGG pathways suggest their involvement in metabolic pathways (Figure 8B).

### 3.7 Expression profiling of *AhCRKs* under drought and salt stresses

**FIGURE 9:**
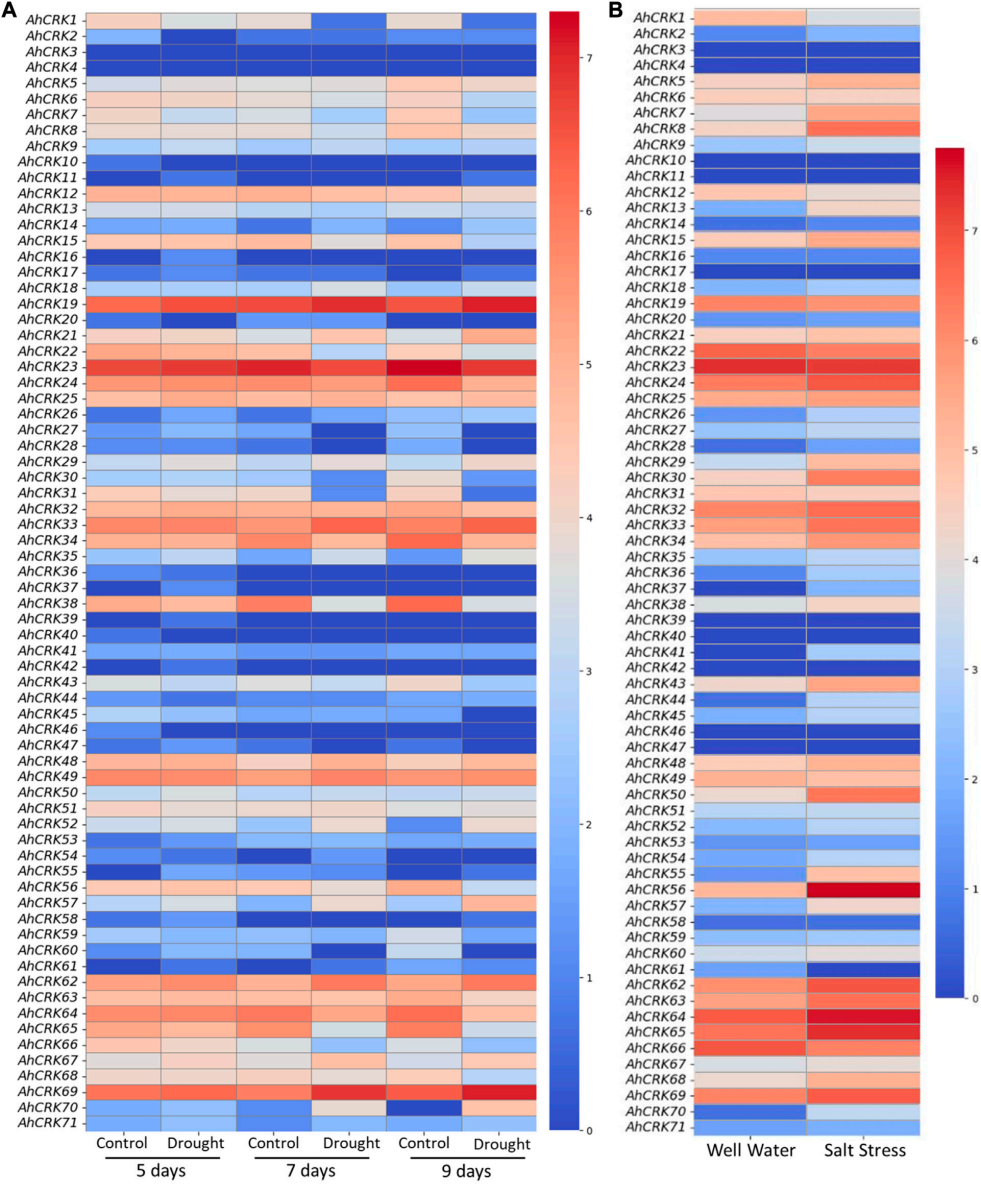
Heatmap representing the change in the expression level of *AhCRKs* in peanut leaves under **(A)** drought stress at 5, 7, and 9 days and in **(B)** salt stress. Blue color represents the downregulated expression, and red color represents the higher or upregulated expression.

Transcriptome expression data were used in the determination of the expression level of 71 *AhCRKs* in leaf tissues under drought and salt stresses. *AhCRKs* possessed diverse expressions under different stress conditions. Under drought stress conditions, most of the genes were highly expressed including *AhCRK19*, *AhCRK22*, *AhCRK23*, *AhCRK24*, *AhCRK25*, *AhCRK32*, *AhCRK33*, *AhCRK34*, *AhCRK38*, *AhCRK48*, *AhCRK49*, *AhCRK62-65*, *AhCRK67*, and *AhCRK69*. All these genes had the same expression under all control and treated conditions, while *AhCRK1*, *AhCRK29*, *AhCRK30*, *AhCRK31*, *AhCRK57*, and *AhCRK70* had fluctuating expression under control and treated conditions (Figure 9A). In salt stress, an almost similar expression pattern of *AhCRKs* was observed as in drought stress, whereas genes including *AhCRK1*, *AhCRK13*, *AhCRK29*, *AhCRK41*, *AhCRK55, AhCRK56*, *AhCRK57*, and *AhCRK68–70* had changed expression under control and treated conditions (Figure 9B).

### 3.8 Identification and performance evaluation of multi-stress responsive genes with a classification algorithm

Among all the differentially expressed *AhCRK* genes, three genes *AhCRK8* (Group I), *AhCRK23* (Group III), and *AhCRK57* (Group II) were found to be common in both drought and salt datasets. After the identification of these potential multi-stress responsive genes, a machine learning classifier, random forest, was implemented to evaluate the performance of these multi-stress responsive genes. To perform this task, the count data of salt stress were provided as a training dataset and multi-stress-related genes (*AhCRK8*, *AhCRK23*, and *AhCRK57*) were tested (Table 2). The ROC plots took into account the sensitivity, the specificity, and the false positive rate (FPR). Sensitivity = True Positives/(True Positives + False Negatives), the proportion of the actual positives that have been correctly identified by the classification model, and the specificity = True Negatives/(True Negatives + False Positives) and the FPR which is the measure of accuracy of the test; Accuracy = (True Positives + True Negatives)/(True Positives + False Positives + True Negatives + False Negatives). The ROC values observed for *AhCRK8*, *AhCRK23*, and *AhCRK57* were 0.6667, 0.8333, and 0.5556, respectively. *AhCRK23* was found to have acceptable ROC values, thus making it a potential multi-stress responsive gene. Supplementary Figure S7 shows the ROC plots for these genes.

**TABLE 2:** Summary of common DEGs identified in salt and drought stress.

Gene symbol	Drought		Salt	
	log2FC (0.5)	*p*-value (<1)	log2FC (0.5)	*p*-value (<1)
*AhCRK8*	−1.275	0.0005	−2.004	0.008
*AhCRK23*	−0.504	0.0245	0.6419	0.026
*AhCRK57*	−1.354	0.0356	−2.0652	0.008

### 3.9 3D structure prediction of AhCRK proteins

**FIGURE 10:**
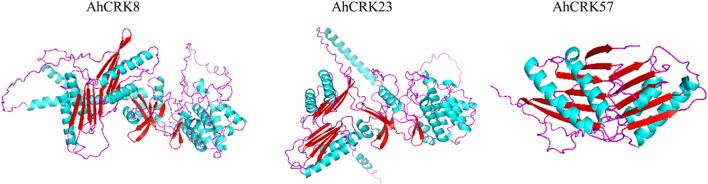
Predicted 3D structures of three multi-stress-related AhCRKs. Structures are displayed based on secondary structures: blue colors represent spirals, red shapes represent turns, and purple shapes represent loops.

To obtain more structural and ultimately functional insights, the 3D protein structures of three multi-stress-related AhCRKs were modeled. The AhCRK8 and AhCRK23 had almost similar structures. The long spirals can be seen in both very similar structures. Similarly, the turns and loops also share similar patterns in both structures. However, the structure of AhCRK57 is significantly different, having a fewer number of helices than those of AhCRK8 and AhCRK23 with a similar number of turns and loops. The predicted similar structures suggest the potentially similar functions of these AhCRK proteins (Figure 10).

## 4 Discussion

RLKs are perceivers of a variety of external environmental stimuli and transmit these input signals to activate the expression of specific target genes (Morris and Walker, 2003). RLKs contain an extracellular domain, an amino-terminal signal sequence, a cytoplasmic domain with serine/threonine protein kinase activity, and a single transmembrane domain. RLKs have several sub-families based on conserved residues; one of them is cysteine-rich repeat (CRR) RLKs (CRKs) (Czernic et al., 1999). CRKs have crucial roles in plants’ immunity, defense, and response to ultraviolet radiation and abiotic stresses (Liu et al., 2021). The CRK family has multiple members in different species with 44 members identified in *A. thaliana* (Chen et al., 2004), 36 in rice (Shumayla et al., 2019), 36 in *Malus domestica* (Zuo et al., 2020), and 30 in *Gossypium barbadense* (Li et al., 2018). However, there is no detailed study available evaluating the intraspecies diversity in *Arachis* members. The current study is being carried out on the CRK family in three *Arachis* species, namely, *A. hypogaea*, and its progenitors *A. duranensis* and *A. ipaensis*.

In our study, we identified 71, 36, and 44 *CRK* genes in *A. hypogaea*, *A. duranensis,* and *A. Ipaensis*, respectively. They exhibit nearly consistent molecular weight, length of amino acids, isoelectric point, aliphatic index, instability index, and GRAVY values with DUF26 and receptor-like kinase domain conserved in them.

All the *Arachis* CRKs have been precisely studied to understand their phylogenetic relationships. The difference in the number of CRKs across *Arachis* members, specifically *A. hypogaea* having 71 members and the other two members containing almost half the number of CRK proteins, shows their ancestral relationships. *A. hypogaea* originated through the combination of both *A. duranensis* and *A. Ipaensis* genomes and have double the number of chromosomes as present in each progenitor. All the CRK members are clustered on the three clades, with each clade containing *Arachis* and *A. thaliana* members. Various members of *A. thaliana* are shown to have roles in stress mechanisms. *AtCRK1* positively regulates the heat and shock responses. *AtCRK5* are the potential regulators of responses to various environmental stimuli (Baba et al., 2018). Similarly, overexpression of the *CRK5* gene altered the defense and growth responses in transgenic plants (Chen et al., 2004). In *O. sativa*, *OsCRK1, OsCRK3*, and *OsCRK4* were expressed in different developing stages, indicating the involvement of these genes in development mechanisms (Yadav et al., 2020). The homology and these genes with *Arachis* members suggest that they also have similar functional conservation and evolutionary significance.

The gene structure, number of intron–exons, and motifs can reflect the expansion, evolution, and functional relationships among the members of the gene family. These are caused mainly by three different types of mechanisms: gain/loss of intron/exon, insertion/deletion, and exonization/pseudoexonization (Wei et al., 2019). The pattern of the gene structure and motif number of CRK members is consistent across various species, indicating they are well-conserved during evolution. A similar pattern of the gene structure was observed in *Arachis* species and others, including watermelon (Wei et al., 2019), chili pepper (Rout et al., 2022), and *Brassica napus* (Sarwar et al., 2023). However, the number of introns and exons varied among the members belonging to different groups. The motif number was also highly conserved among members across CRK members from various species, such as in *B*. *napus* (Sarwar et al., 2023), hot pepper (Srideepthi et al., 2020), and in *P. vulgaris* (Quezada et al., 2019). Some members such as *AhCRK42*, *AdCRK36*, *AdCRK5,* and *AdCRK9* did not contain any intron, which indicates early evolution as well, and they might have active involvement in plant development and metabolism (Chakraborty et al., 2023). All the variation in exon and intron numbers across *Arachis* and other species suggests the evolution of gene structures over time, which ultimately affects their functional conservation. This indicates the diversification of *CRK* genes. Since almost all the genes had the similar number of motifs conserved in them, it shows that their functions remained conserved during evolution. According to phylogeny analysis, it seems that CRK family members have been affected by evolutionary events that have caused their expansion, although functional diversity was observed between them. Probably, the modifications in the gene structure and regulatory regions of genes during evolution have caused the diversity of expression between members of a gene family. However, further studies are needed to understand the possible role of these modifications (Hashemipetroudi et al., 2023; Yaghobi and Heidari, 2023).

The chromosomal localization was relatively conserved among *Arachis* members, and Chr7 had no gene in all three Arachis species. However, the gene numbers clustered at almost the same chromosomal sites slightly varied. Evolutionary patterns are attributed to the mechanism of duplication of genes, including segmental and tandem. Segmental duplications are highly prevalent in plants, particularly in diploidized polyploids, where multiple duplicated chromosomal segments are retained, contributing to the abundance of duplicated genomic blocks within their genomes (Quezada et al., 2019). Both tandem and segmental duplication have played a significant role in the expansion of the CRK gene family. However, most plant species exhibited segmental duplication across their genomes (Wei et al., 2019; Zhao et al., 2021). However, in *A. hypogaea*, most *CRK* genes were segmentally duplicated, whereas *A. duranensis* and *A. ipaensis CRKs* observed tandem duplication. The determination of selection pressure on any protein or gene was done by utilizing the Ka/Ks ratio, where the mutation ratio was utilized. Ka/Ks greater than 1 represents positive selection, while Ka/Ks less than 1 shows purifying selection. *Arachis* species showed both positive and purifying selection.

*Cis*-regulatory elements are also one of the key players in regulating the stress-responsive activities of *CRK* genes and act as molecular switches, thus regulating gene expression. The *cis*-elements identified in the *CRK* gene family are related to defense-related, hormone, and abiotic stress-responsiveness. Expression analysis studies showed the involvement of *AhCRK* genes in drought and salt stress, which is evident by the presence of stress response elements in their promoters. Similarly, in *Capsicum annuum* (pepper), *CaCRK5* is involved in a mechanism related to the immune response against pathogens. Various elements in this gene’s promoter region contributed to this defense response (Mou et al., 2021). The *Triticum aestivum* gene *TaCRK68-A* showed its recombinant expression in *Saccharomyces cerevisiae* and *Escherichia coli*, thereby enhancing their tolerance against drought, salinity, cold, and heat stress (Shumayla et al., 2019). This could be speculated that *AhCRK* genes’ expression is promoted by abiotic stresses, although further work is required to confirm this. miRNAs have received significant attention for their roles in stress tolerance and development. We identified miRNAs belonging to multiple families which targeted *AhCRK* genes. Other peanut genes involved in abiotic stress responses have also found miRNAs targeting them, thereby controlling their expression levels (Cai et al., 2023). The GO analysis of *AhCRK* genes exhibited their distinct roles in external stimulus and defense response and their involvement in functions like kinase activity. Previous studies reported the *CRK* genes’ roles in the positive regulation of stress responses, thus showing their involvement in various metabolic and biological pathways (Shumayla et al., 2019). The PPI analyses of these genes also showed their interaction with the other proteins involved in kinase- and stress-related activities.

The expression profile of *CRK* genes correlated with two abiotic stresses: drought and salt treatment in *A. hypogaea*. The degree of upregulation and downregulation varied in both stresses. In drought stress, almost half of the genes were upregulated (*AhCRK23* and *AhCRK69*), and the others were downregulated, whereas some genes showed a change in expression on different days of tolerating drought stress, including *AhCRK1, AhCRK21*, *AhCRK38*, *AhCRK56*, *AhCRK57*, and *AhCRK70*. This indicates their crucial roles in drought stress tolerance. Moreover, in salt stress, the expression also varied under normal and treated conditions. Some genes were highly upregulated or downregulated upon exposure to salt stress (*AhCRK1*¸ *AhCRK7*, *AhCRK8*¸ *AhCRK23*, *AhCRK56*, *AhCRK68*, *AhCRK69*, and *AhCRK70*). Similar results were observed in other plants under abiotic stresses, including cold, salt (Zhang H. et al., 2017), heat, and drought (Shumayla et al., 2019). This shows that these genes would have important stress regulatory roles in real-world scenarios and fortify the foundation for future crop improvement strategies. Furthermore, the machine learning approaches were used to evaluate the genes which showed co-expression in both drought and salt stress. Three genes *AhCRK8*¸ *AhCRK23*, and *AhCRK57* were found to show responsiveness under multi-stress-related conditions. The 3D structures of these three proteins were also predicted to help understand their structural and functional conservations. Hence, it can be inferred that *CRK* genes are regulated in abiotic stresses and help plants thrive under those conditions. In future studies, leveraging natural genetic variation within the germplasm to validate the function of identified candidate genes under specific stress conditions holds a significant confirmation value. These perspectives could contribute valuable insights toward the selection and integration of these genes in breeding and genetic engineering initiatives to enhance stress resilience in crops. Thus, these genes, most importantly the multi-stress responsive genes, can be used in future research studies on peanut.

## 5 Conclusion

CRKs are found to have regulatory roles in plants under various abiotic and biotic stresses. This study provides not only a systematic but also a comparative analysis of *CRK* genes in three nutritious and economically important peanut species. A total of 71, 36, and 44 genes were identified in *A. hypogaea*, *A. duranensis,* and *A. ipaensis*. The results elucidate the structural and physiochemical properties of the CRK gene family, which shows the intraspecies diversity and evolutionary conservation. The results also provide deep insights into the roles of *CRK* genes in the development, growth, environmental stimuli, and the mediation of abiotic stresses (salt and drought). *AhCRK19*, *AhCRK23*, *AhCRK56*, and *AhCRK69* can potentially be candidate genes for conferring tolerance against drought stress. On the other hand, *AhCRK8*, *AhCRK23*, *AhCRK24*¸ *AhCRK56*, *AhCRK65,* and *AhCRK69* can act as potential candidate genes in providing resistance against salt stress. Machine learning approaches were utilized to evaluate the multi-stress responsiveness of these genes. Owing to their expression on both drought and salt stress, *AhCRK8*, *AhCRK23*, and *AhCRK57* can be deemed candidate genes for multi-stress responsiveness. These genes are needed to be explored further and can be used in genetic engineering research to devise multi-stress-resistant and -tolerant crops. Our study will also help further investigate the functional roles of the *CRK* genes in peanuts.

## Data Availability

The original contributions presented in the study are included in the article/Supplementary Material, further inquiries can be directed to the corresponding author.
