# Supplementary material for: Integrated omics and machine learning-assisted profiling of cysteine-rich-receptor-like kinases from three peanut spp. revealed their role in multiple stresses
Source: Front Genet. 2023 Sep 20;14:1252020. doi: 10.3389/fgene.2023.1252020 (PMC10547876; doi:10.3389/fgene.2023.1252020)
Supplement: Supplementary file 2 [file Table1.DOCX]

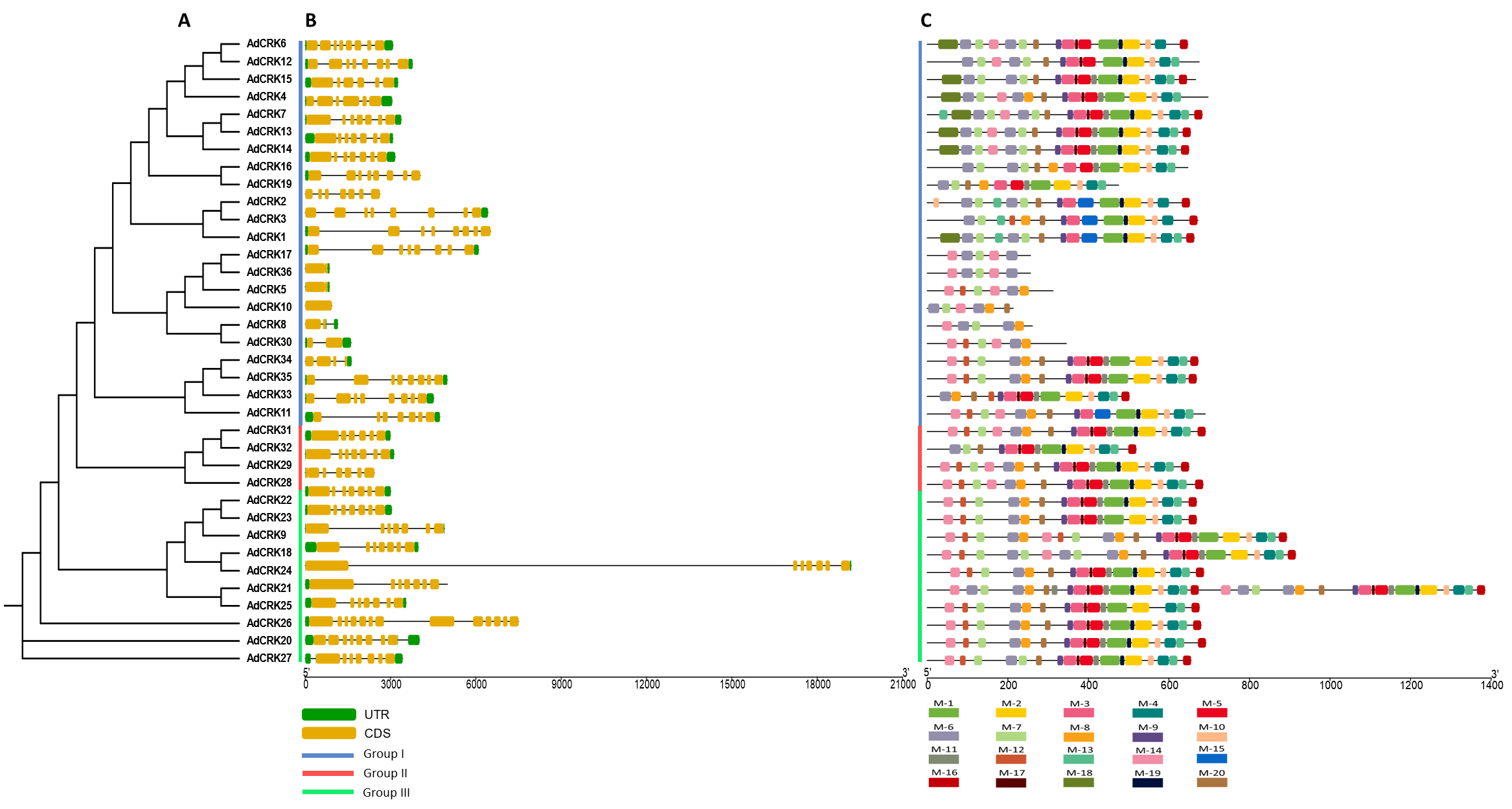


**Figure S1: (A)** Phylogenetic tree of AdCRKs, **(B)** structural features showing exon-intron organization, and **(C)** conserved motif pattern of 36 AdCRKs proteins.


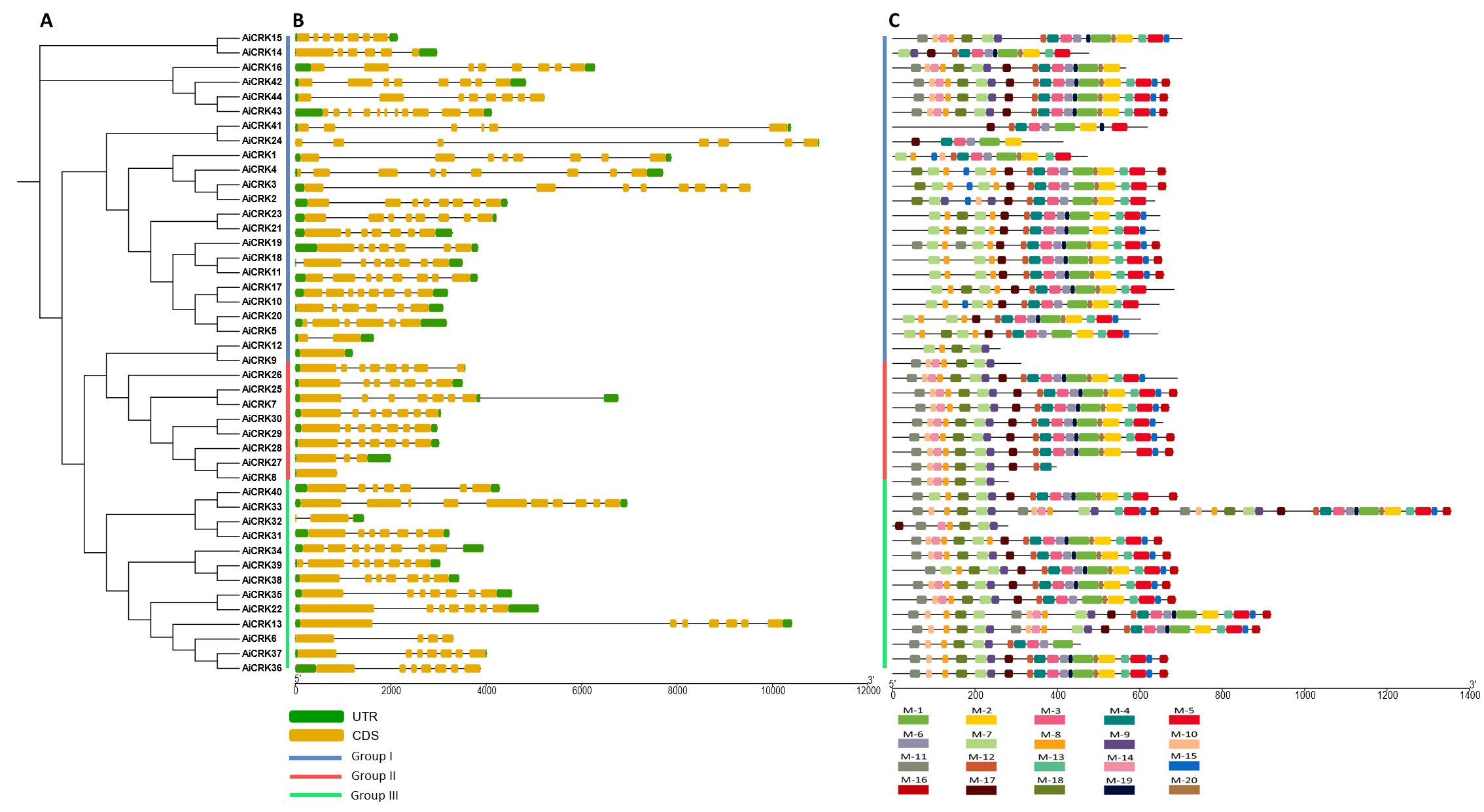


**Figure S2: (A)** Phylogenetic tree of AiCRKs, **(B)** structural features showing exon-intron organization, and **(C)** conserved motif pattern of 44 AiCRKs proteins.


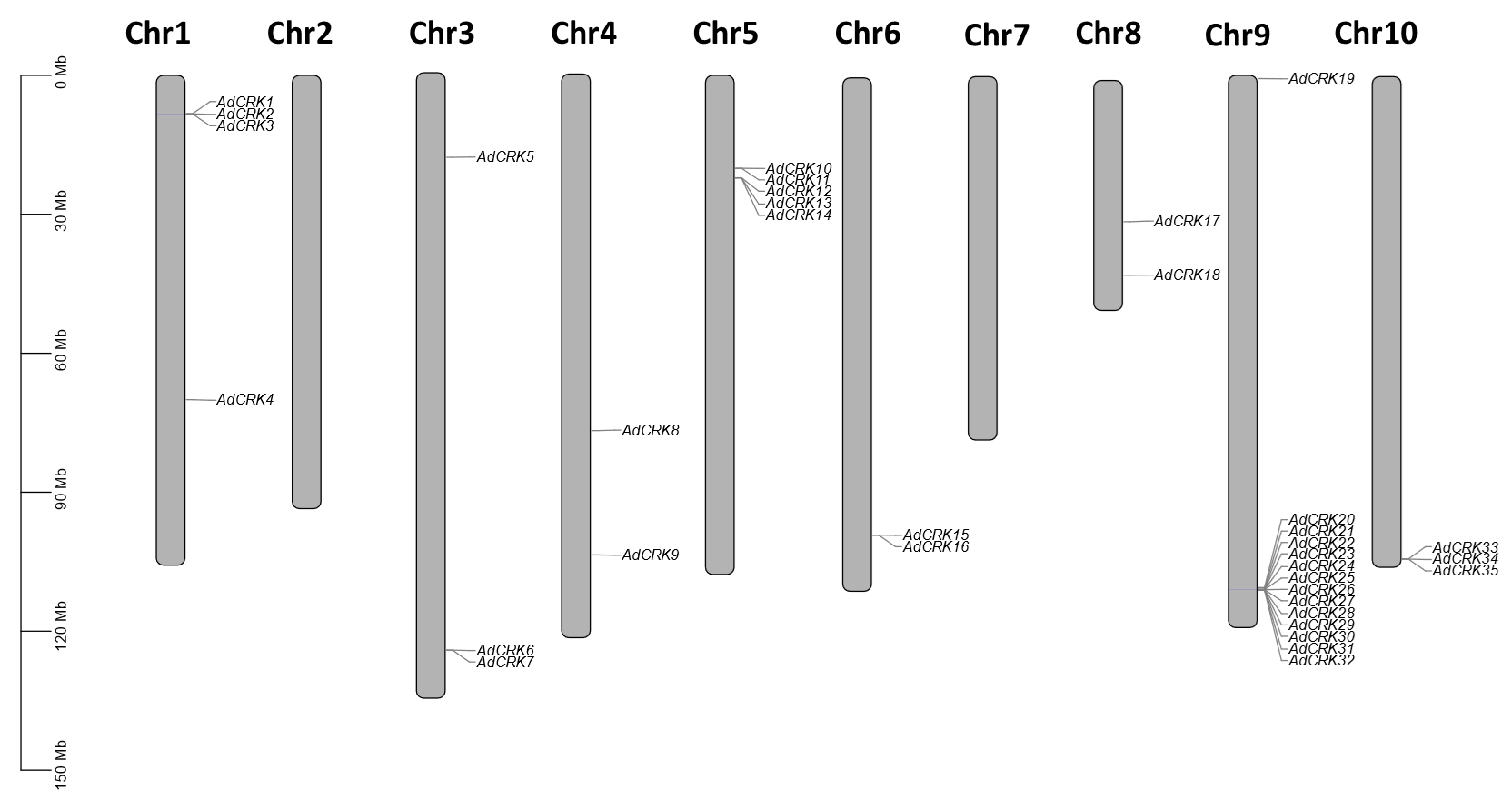


**Figure S3:** Chromosomal mapping of *AdCRK* genes.

**
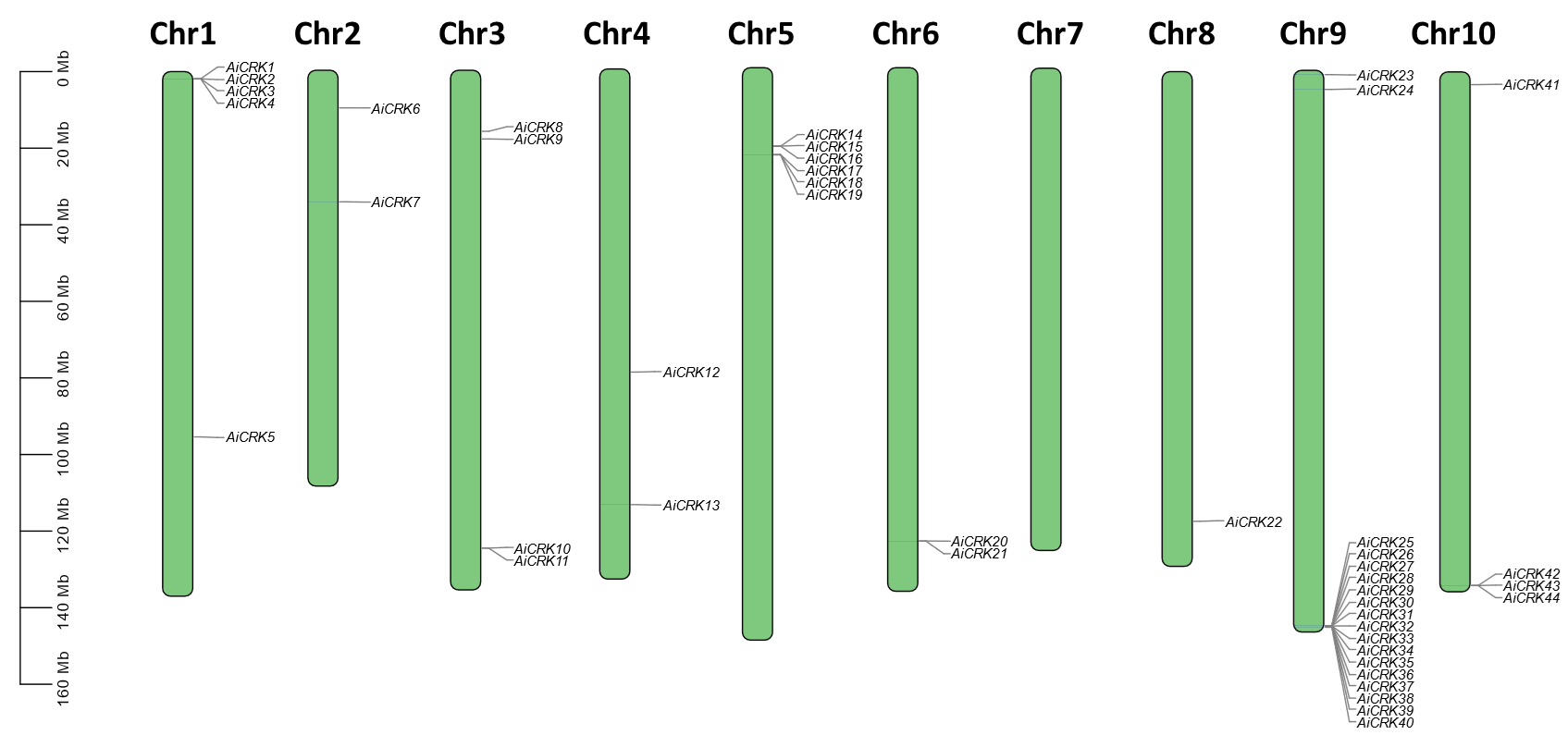
**

**Figure S4:** Chromosomal mapping of *AiCRK* genes.


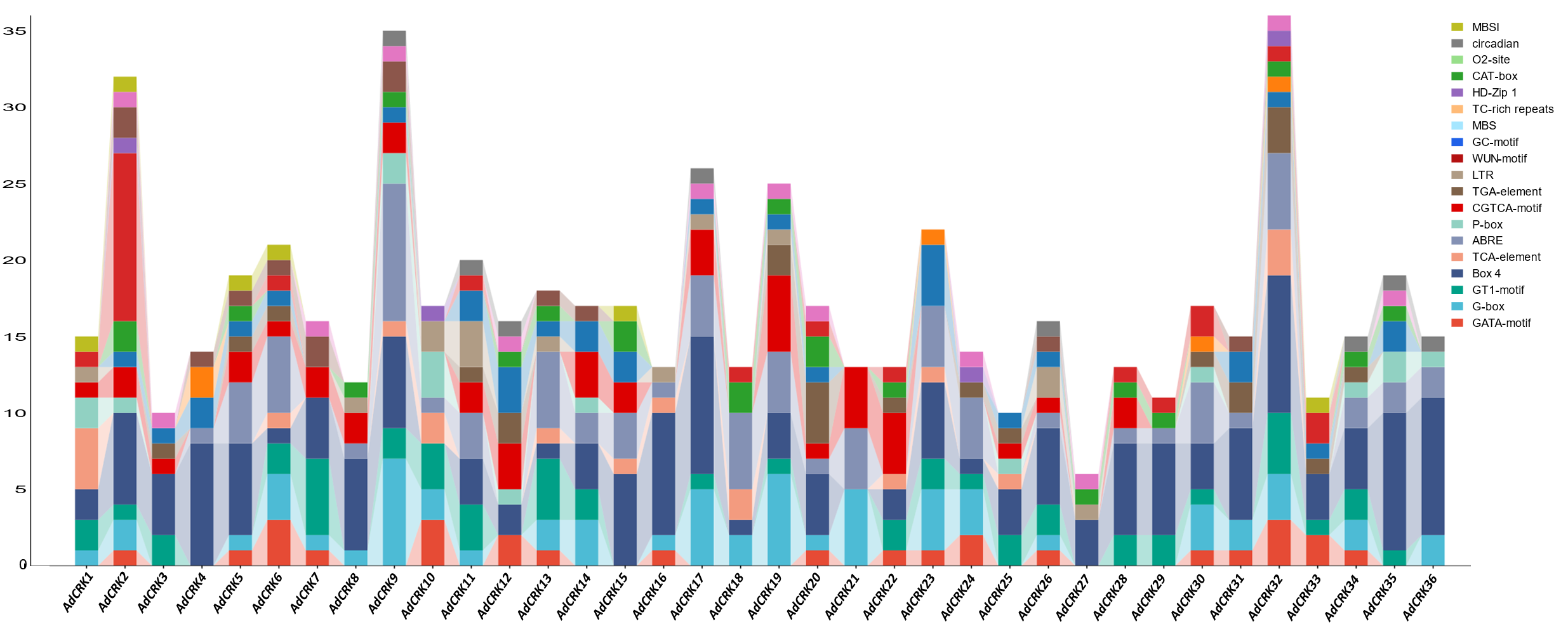


**Figure S5:** Cis-regulatory elements in the promoter region of *AdCRK* genes. Each bar is representing the specific elements present in the particular gene.


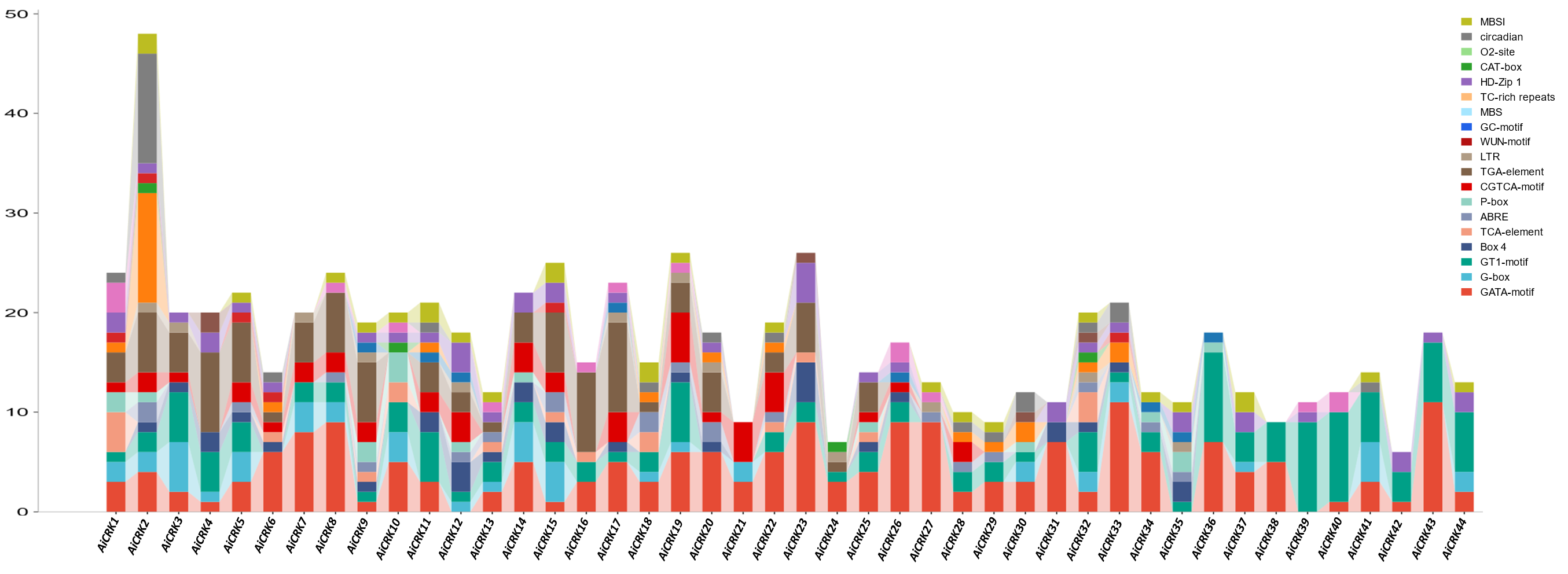


**Figure S6:** Cis-regulatory elements in the promoter region of *AiCRK* genes. Each bar is representing the specific elements present in the particular gene.


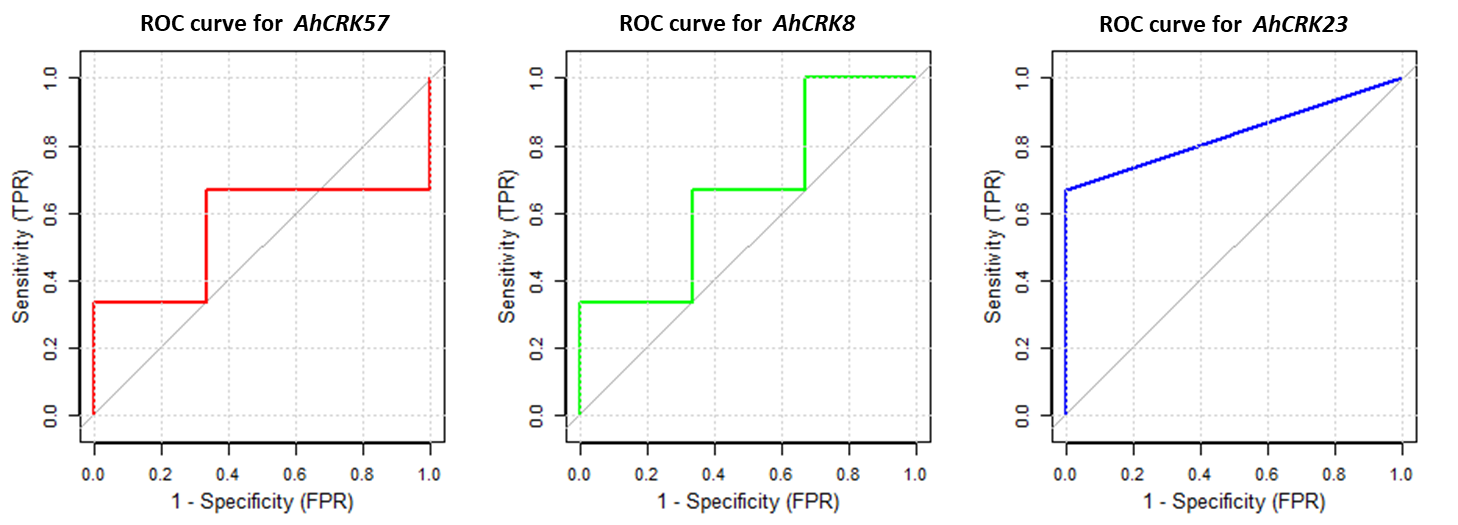


**Figure S7:** Classification performance of random forest classifier, Receiver operating curve (ROC) plot of the classifier based on the sensitivity, specificity, and accuracy.
